# Tears and saliva as alternative matrices for minimally invasive assessment of acute stress and pain in sheep

**DOI:** 10.3389/fvets.2026.1719442

**Published:** 2026-01-28

**Authors:** Fanny Rachidi, Romy Wagner, Helena Fieseler, Matthias Kaiser, Hendrik Müller, Lilli Bittner-Schwerda, Ožbalt Podpečan, Norbert Mielenz, Jutta Gottschalk, Almuth Einspanier, Gerd Möbius, Alexander Starke

**Affiliations:** 1 Clinic for Ruminants and Swine, Faculty of Veterinary Medicine, Leipzig University, Leipzig, Germany; 2 Farm Animal Practice VM- Striegistal, Striegistal, Germany; 3 National Center for Animal Welfare, Faculty of Veterinary Medicine, University of Ljubljana, Ljubljana, Slovenia; 4 Biometrics and Informatics in Agriculture Group, Institute of Agricultural and Nutritional Sciences, Martin-Luther-University Halle-Wittenberg, Halle, Germany; 5 Faculty of Veterinary Medicine, Institute of Physiological Chemistry, Leipzig University, Leipzig, Germany; 6 Faculty of Veterinary Medicine, Institute of Animal Hygiene and Veterinary Public Health, Leipzig University, Leipzig, Germany

**Keywords:** acute stress response, animal welfare, claw treatment, cortisol, dorsal recumbency, lacrimal fluid, stress memory, tilt squeeze chute

## Abstract

This study evaluated tears and saliva as alternatives to blood for determining cortisol concentration in healthy and chronically lame Merino meat sheep with dermatitis interdigitalis contagiosa (DINCO). Twelve healthy (HEALTHY) and 36 sheep with DINCO were included. After enrollment and placement of a jugular vein catheter on day 0, sheep underwent daily clinical examination and tear, saliva, and blood sampling on days 1 to 6 to determine cortisol concentrations (tears: COT; saliva: COS; blood: COB). After a 4-day adaptation period, blood and tears were collected six times at defined intervals immediately after the application of standardized stressors during a 1-h treatment phase. Sheep with DINCO were randomly allocated to one of three treatment groups and underwent claw treatment with or without pain control: the XYLA-IVRA group received sedation and retrograde intravenous regional anesthesia (IVRA); the IVRA group underwent IVRA and received a placebo instead of sedation; the PLACEBO and HEALTHY groups received isotonic saline instead of sedation and IVRA. The HEALTHY group underwent sham claw treatment. Statistical analyses used linear mixed models (PROC MIXED, SAS 9.4). Saliva and tear collection was minimally invasive, repeatable, and consistently yielded sufficient material for analysis without adverse health effects. Cortisol was detected in saliva and tears at all time points, and concentrations did not differ between healthy and diseased sheep during the adaptation phase. Both matrices had lower concentrations than blood but correlated positively and significantly with each other (day 2, *R* = 0.86 ± 0.05) and with COB (R_COB−*COS*_ = 0.71 ± 0.1, R_COB−*COT*_ = 0.61 ± 0.1). During the treatment phase, the COT increased in response to stressors similar to COB, albeit with a median delay of 6 min. The COT tended to be higher in the PLACEBO than in the XYLA IVRA group. Considering the time delay, saliva and tears are reliable, minimally invasive collected alternatives to blood for determining cortisol concentrations and assessing an acute stress response in healthy and chronically lame sheep.

## 1 Introduction

Acute stress is a critical challenge in both human and veterinary medicine, especially during clinical and standard care procedures. Stressors can induce physiological stress responses that, depending on the individual's experience and perception, may negatively impact health and welfare (1–3). Similar to human neonates and those with cognitive impairment, animals are unable to verbally communicate stress or pain levels, although they may experience stress to a similar extent (4–7). This underscores the need for objective methods to detect acute stress responses independent of self-reported symptoms.

Sheep are commonly used in veterinary and translational research because of their physiological similarities to humans in overall stature, weight, and wound healing (8, 9). However, as a prey species with a strong flight instinct, sheep may actively mask or dissimulate behavioral signs of distress, complicating clinical recognition and increasing the risk of undetected stress (10). This not only raises concerns for animal welfare but may also compromise data quality and interpretation. Consequently, validated, species-specific stress models have been developed, including procedures such as restraint, isolation, transport, shearing, and hoof trimming (11–15). These models are also essential for developing and refining stress detection, evaluation of findings, and mitigation strategies in human medicine, where such approaches are often tested in animal experiments before clinical translation (16, 17).

Stress in sheep can be assessed using behavioral, clinical, and physiological markers. Among the latter, the quantification of blood cortisol concentration (COB) is considered the gold standard for measuring an acute stress response (18). However, blood collection is inherently invasive, requiring restraint and venipuncture, which may be painful, cause complications such as thrombophlebitis, and potentially induce a stress response that interferes with the validity of stress measurements (19, 20).

To mitigate the confounding effects of invasive sampling, alternative matrices and less invasive techniques have been investigated. One approach is blood collection via indwelling venous catheters, which allows repeated sampling without repeated venipuncture, but still involves invasive procedures (15, 20). Alternatively, secondary matrices, in which cortisol or its metabolites can be detected, have been studied as minimally invasive and non-invasive alternatives (21–23). In human medicine, such methods are considered promising for continuous stress monitoring. For instance, cortisol concentration in tears (COT) has been measured using wearable biosensors (24, 25); however, standardized protocols and clinical validation are lacking.

In sheep, cortisol determination has been reported for several matrices, including feces, saliva, urine, milk, wool, and tears (20, 23, 26–28). Nevertheless, not all matrices are suitable for acute stress assessment. Fecal and wool cortisol concentrations are limited by delayed metabolite excretion (22, 29), and matrices such as milk, urine, and wool are either inconsistently accessible or prone to contamination (21, 30, 31). In contrast, saliva and tears offer a more practical and timely alternative (21, 32). Only free, biologically active cortisol diffuses from the blood into these matrices within a few minutes and therefore directly reflects the acute stress response (26, 33–36). Both matrices can be analyzed using the same radioimmunoassay as applied for blood cortisol analysis and do not require extensive pre-analytical decontamination protocols (15, 20, 21, 31). However, saliva exhibits high, individually variable bacterial loads and may be contaminated with blood or inflammatory products due to mucosal lesions (37, 38). Additionally, regurgitated cud poses a contamination risk in ruminants (20). Consequently, continuous saliva sampling for cortisol analysis via intraoral capsules has not become established (39, 40). Conversely, in healthy individuals, tears demonstrate significantly lower bacterial loads and contamination risk (41). Continuous monitoring via wireless contact lenses has already been developed and represents a potential application for continuous cortisol monitoring in human medicine (24, 25). While the cortisol concentration in saliva (COS) is an established stress marker in sheep (42), the suitability of tears, particularly in clinically affected animals, has not yet been validated (PubMed search on December 18, 2025, keywords: sheep, cortisol, tears).

The present study aimed to evaluate the suitability of tears and saliva collected by minimally invasive methods for determining cortisol concentrations as alternatives to blood for assessing acute stress and pain in healthy and Merino meat sheep with DINCO.

## 2 Material and methods

This study was part of a larger project focusing on pain management and individual stress response during treatment of DINCO in ewes. In addition to the data presented here, behavioral and physiological data were collected during the study (10, 43). The study was approved by the State Administration Office (Landesverwaltungsamt, Referat Verbraucherschutz, Veterinärangelegenheiten Sachsen-Anhalt, Germany); permit number 42502-3-734. This randomized, prospective, triple-blinded study was carried out on a commercial sheep farm with 700 Merino meat ewes in central Germany between December 2014 and May 2015.

### 2.1 Animals

**Figure 1:**
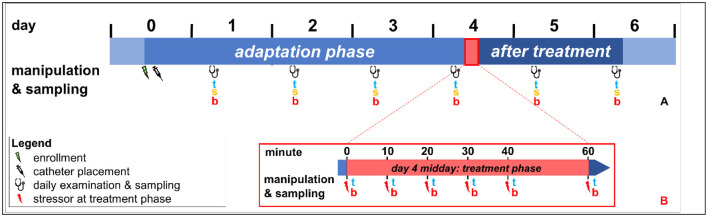
Illustration of the experimental design; **(A)** Daily handling of the study sheep (clinical examination of standing sheep in the morning; remaining manipulations in dorsal recumbency): Day 0: Enrollment and complete physical examination, placement of jugular vein catheter, and trimming of both front feet and one hind foot. Midday of day 0 to midday of day 4: adaptation phase. Day 4 after treatment phase to midday of day 6: post-treatment phase, clinical examination and sampling of tears (t), saliva (s) and blood (b; from jugular vein catheter). Day 6: end of the study, removal of the jugular vein catheter. Day 4 is part of the adaptation phase, the treatment phase, and the post-treatment phase. **(B)** Manipulation and tear and blood sampling during the treatment phase in a tilt squeeze chute on midday of day 4: Min 0: sheep is placed in tilt squeeze chute and receives medication according to group allocation; Min 10: positioning of the sheep in dorsal recumbency; Min 20: administration of IVRA or placebo according to group allocation; Min 30: claw treatment, actual or sham; Min 40: resumption of standing position; Min 60: sampling in group pen.

Forty-eight female merino meat sheep were selected and included in the present study. The ewes were an average of 2.4 ± 1.9 years of age (1.1–8.4 years, mean ± standard deviation (SD) [min–max]), weighed an average of 53.8 ± 9.1 kg (39.5–78.0 kg, mean ± SD [min–max]), and had a median body condition score (BCS) of 3.25 ± 0.3 of 5 (2.00–3.75, mean ± SD [min–max]). Twelve sheep met the inclusion criteria for the HEALTHY group (normal demeanor, less than 100 days pregnant, no antibiotic or antiphlogistic treatment). The remaining 36 sheep fulfilled the criteria for the DISEASED group (same inclusion criteria as for HEALTHY sheep, except *dermatitis interdigitalis contagiosa* (DINCO) was present in one hind foot). The study design was similar to that used by Wagner et al. and Rachidi et al. (Figure 1). Twelve study groups, each consisting of four sheep (three diseased and one healthy), were consecutively and randomly formed; all sheep entered the study at the same time. At any time, only one group of four sheep was studied, and the next group was formed only after data collection from the previous group had been completed.

**Figure 2:**
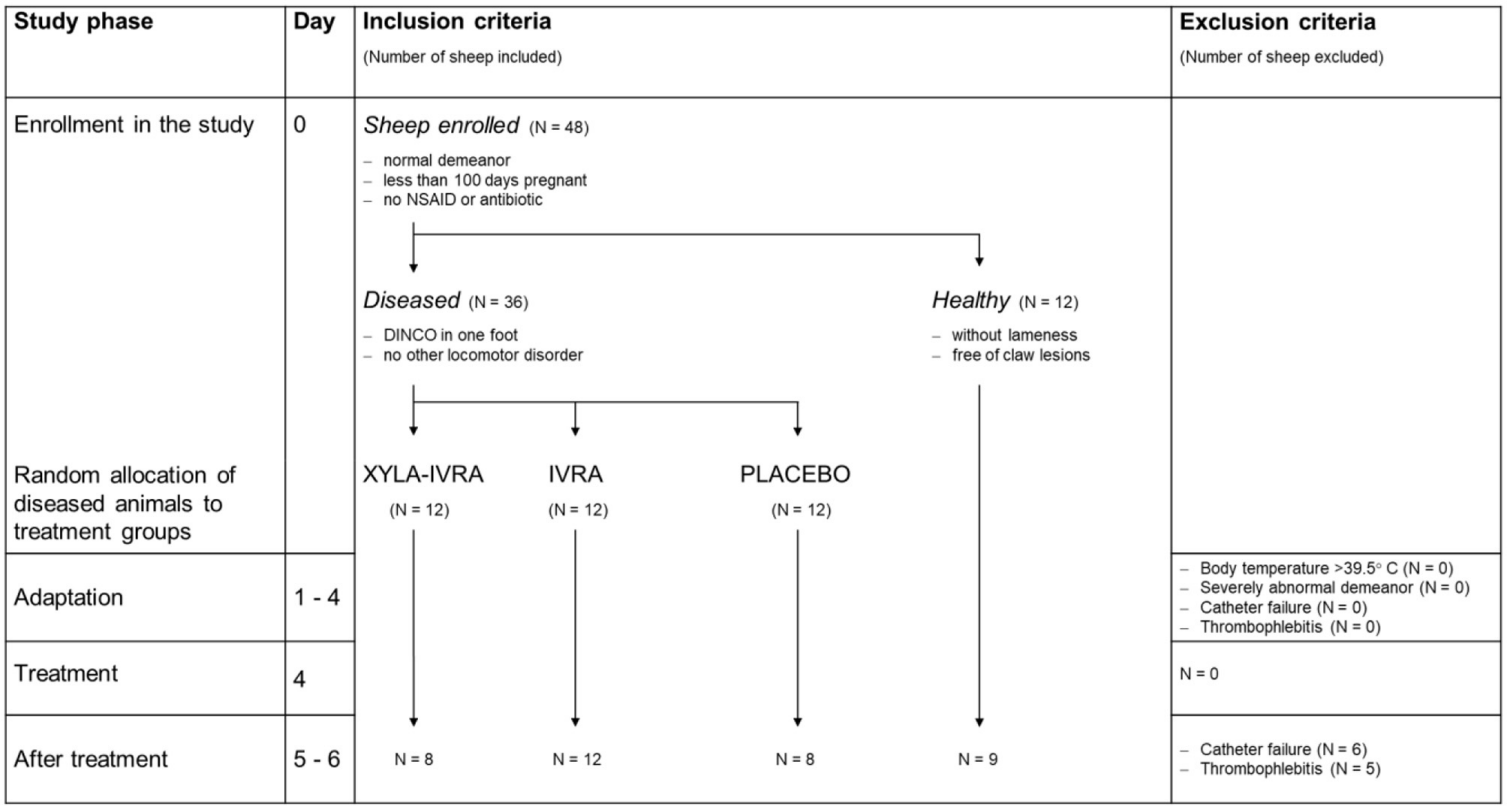
Study workflow with inclusion and exclusion criteria, enrollment of 36 sheep with *dermatitis interdigitalis contagiosa* (DINCO; Diseased) and 12 healthy sheep (Healthy), random allocation of the diseased sheep to the treatment groups (XYLA-IVRA = sedation and local anesthesia, *n* = 12; IVRA = local anesthesia, *n* = 12; PLACEBO, *n* = 12), exclusion of 11 sheep after treatment (day 5 and 6).

The criteria for inclusion and exclusion of sheep from the study were described in detail by Rachidi et al. To confirm the flock diagnosis of DINCO, all sheep on the farm were checked daily throughout the study. Suitable animals (*N* = 48) were enrolled in the study based on the inclusion criteria. Exclusion of study sheep was not necessary, but in 11 of the 48 study sheep, in which either continuous blood sampling via the jugular vein catheter was no longer possible or thrombophlebitis developed on day 5 or 6, cortisol values from days 5 and 6 were excluded from the analysis (Figure 2).

To confirm the endemic infection of the flock with *Dichelobacter nodosus*, swab samples were collected from the interdigital space of five sheep with characteristic clinical signs before, during, and after the study. The five sheep did not fulfill the inclusion criteria and were chosen to avoid exposing the study sheep to the stress of sample collection. The samples were examined by PCR for the virulent strain of *Dichelobacter nodosus* (Food Safety and Animal Health Office, Chur, Switzerland), and all were positive.

### 2.2 Study design

Housing and handling of the sheep were described previously (15). Analgesia for treatment of the claw lesions in the DISEASED group was done randomly using a lottery system, as described by Schären et al. (81):

XYLA-IVRA: retrograde intravenous regional anesthesia (IVRA) (10) using 5 ml of 2% procaine hydrochloride (Procasel- 2%^®^, Selectavet Dr. Otto Fischer, Germany) and sedation with 0.1 mg/kg of 2% xylazine hydrochloride (Rompun^®^, Bayer Vital, Germany) administered intramuscularly.

IVRA: IVRA using 5 ml of 2% procaine hydrochloride and an equivalent volume of isotonic saline solution (isotonic sodium chloride solution 0.9% Braun^®^, B. Braun Melsungen AG, Germany), administered intramuscularly, instead of xylazine hydrochloride.

PLACEBO: 5 ml isotonic saline solution, administered intravenously, instead of procaine hydrochloride for IVRA, and an equivalent volume of isotonic saline solution, administered intramuscularly, instead of xylazine hydrochloride.

HEALTHY: received the same treatment as the PLACEBO group.

At the start of the study (day 0), the sheep underwent a thorough physical examination, including an orthopedic examination while standing and walking 10 m on a level surface. After this, the sheep of each group were led individually to a tilt squeeze chute to assess posture, behavior, physical condition, fleece, body condition, respiratory rate, pulse rate, and body temperature. Sheep in the HEALTHY group were tilted into dorsal recumbency, the limbs were secured, and both front feet and the left hind foot were trimmed (10, 15). In the DISEASED group, both front feet and the healthy hind foot of each sheep were trimmed. The sheep were then moved back to the group pen. Two hours later, the sheep were restrained manually in a standing position in the group pen, and an indwelling teflon catheter was placed in an external jugular vein (VWI Jugularis-Katheter, outer diameter 2.4 mm, length 10 cm, Walter Veterinär-Instrumente e.K., Baruth/Mark) (14).

Day 1 to the midday of day 4 represented the adaptation phase and day 5 and 6 represented the post-treatment phase (Figure 1). From 8.00 to 10.00 a.m. on days 1 to 6, the sheep of a study group were calmly led to the tilt squeeze chute and clinically examined while standing. After this, the sheep were restrained in dorsal recumbency, and tear, saliva, and blood samples were collected (20), always in the same order. From 11:00 a.m. to 3:00 p.m. on day 4, the DISEASED group underwent claw treatment in the previously untreated hind claw with DINCO, using the previously allocated treatment protocol (XYLA-IVRA, IVRA, PLACEBO). The HEALTHY group underwent sham claw treatment in the right hind foot. Careful removal of undermined horn and necrotic tissue was done using curved claw trimming shears and a claw knife to create a clean transition to healthy tissue. Sham treatment of the right hind claws of the HEALTHY group consisted of the careful removal of loose horn to restore the proper claw shape (15). The procedures carried out during this period represented the stress model, hereafter referred to as the treatment phase, and were conducted in a standardized manner as previously described (15). Tear and blood samples were collected immediately after each experimental procedure. Saliva samples were not collected during the treatment phase to prevent additional stress and to comply with the experimental collection schedule. All clinical examinations and sample collections were carried out by the same two veterinarians (RW, HF). Treatment of sheep in the DISEASED group, sham claw treatment, and drug administration were divided between two other veterinarians (MK, HM). The indwelling catheter was removed on day 6. Bandage changes and monitoring of wound healing were done as described by Fieseler et al. (43).

The collection and storage of the samples and the measurement of the cortisol concentrations were done as follows: Tears were collected using a 2-ml syringe fitted with a blunt needle, onto which a 3-cm segment of butterfly catheter tubing (Surflo Winged Infusion Set 21G × 3/4″, 0.8 × 19 mm, length 30 cm, internal volume 0.42 ml; Terumo Europe N.V.) was attached over the rounded tip. The tubing extended approximately 0.5–1 cm beyond the needle tip. A sample volume of approximately 0.1 ml was obtained in < 1 min. For saliva sampling, a gauze swab (Wilhelm Weisweiler GmbH & Co. KG, Münster, Germany) was wrapped around a sponge-holding forceps, gently inserted into the animal's mouth, and held in place for approximately 1 min while the sheep chewed. The swab was then transferred to a 15-ml polypropylene tube (Cellstar^®^, Greiner Bio-One GmbH, Frickenhausen, Germany). Blood samples were collected via the indwelling jugular vein catheter. Samples from multiple study groups were subsequently collected and transported under cooled conditions to the laboratory. Upon arrival, samples were stored at −80 °C unter analysis, which was performed within a few months after completion of the study. In addition, aliquots were retained as retention samples and remain stored at −80 °C. Determination of the COT, COS, and COB was done as described in detail (21), with total cortisol measured in blood and free cortisol measured in tears and saliva using a ^3^H-radioimmunoassay (label/tracer: [1,2,6,7-3H]-cortisol; intraday variation coefficient 6.5%, interday 7.8%; sensitivity 0.5 ng/ml for serum, saliva, and tears) modified according to Abraham et al. (110).

### 2.3 Statistics

#### 2.3.1 Analysis of repeated measurements

Linear models implemented in the MIXED procedure of SAS version 9.4 (SAS Institute 2013, Cary, NC, USA) was used for statistical analysis. Possible environmental or parity effects during the study period were considered by including fixed effects of the study groups in all models. The repeated measurements within an animal were taken into account by using random animal effects and/or by the variance-covariance structure of the residual effects. The following competing structures were tested: compound symmetry (CS), heterogeneous compound symmetry (CSH), first order autoregressive (AR) (1), heterogeneous first order autoregressive (ARH) (1), unstructured (UN), and Toeplitz with two, four, or six bands [TOEP (2), TOEP (4), and TOEP (6)]. The final correlation structure was chosen according to the lowest value of Akaike's information criterion (AIC) based on the REML method. The Shapiro-Wilk test was used to test the studentized residuals in all models for normality. When the data were not normally distributed, they were log-transformed and tested again for normal distribution. Differences between treatment groups within time points (days or min) and differences between time points within treatment groups were analyzed using the Tukey test. The degrees of freedom were approximated according to Kenward and Roger (ddfm = KR in MIXED). Results were considered statistically significant at *p* ≤ 0.05, and a statistical trend was declared at 0.05 < *p* ≤ 0.10.

#### 2.3.2 Stress response during the treatment phase

Polynomial regression was used to analyze the effect of the targeted pain management protocol by describing the dependence of the cortisol concentration on time (min). The regression coefficients were considered to be specific to the treatment groups (XYLA-IVRA, IVRA, PLACEBO, HEALTHY). The degree of the polynomial was determined using the corrected Akaike's information criterion (AICC) based on the maximum likelihood method. To evaluate the model fit, the least square mean (LSM) curves of the regression models were compared visually with trend curves calculated by the procedure LOESS. Animal-specific regression functions (polynomials of second degree) were fitted simultaneously to the observations per min and the following new traits derived (21): area under the curve (AUC) on the interval from min 0 to 60 (divided by 60), maximum cortisol concentration from min 0 to 60 (Ymax), and the time when maximum cortisol concentration occurred (Tmax). Area under the curve, Ymax, and Tmax were analyzed using multifactorial ANOVA. For this purpose, the study and treatment groups were treated as fixed effects in the model. The polynomial model and the models of the three derived traits contained the cortisol concentration at the start of day 4 (before the treatment phase), representing baseline cortisol concentration as a linear covariate. Phenotypic correlations between the cortisol concentrations in the matrices tears and blood were estimated using two-trait models. The estimated phenotypic correlations resulted from the variances in the residual effects and the covariances between the residual effects of different traits. The correlations were estimated using the MIXED procedure with special data preparation by assigning two observations of the matrices to each animal.

#### 2.3.3 Stress response before and after the treatment phase

The factors groups and days, and their interactions, were represented by fixed effects in the models. The observations of the 11 sheep in which blood collection was unsuccessful on days 5 and 6 were treated as missing values. For the analysis of data collected before the treatment phase, the groups XYLA-IVRA, IVRA, and PLACEBO were combined. Three-trait models were used to determine the estimated phenotypic correlations between the matrices tears, saliva, and blood. For each matrix (tears, saliva, and blood), a linear model, with study and treatment groups as fixed effects, was assumed.

## 3 Results

### 3.1 Adaptation phase

During the adaptation phase, COT and COS did not differ among days 1, 2, and 3. The COB was significantly higher on day 1, which was 1 day after placement of the jugular vein catheter and the first contact with the investigators, compared with the remaining days of the adaptation phase (Table 1). Cortisol concentrations in all matrices did not differ between the HEALTHY and DISEASED groups (Supplementary Table 1).

**Table 1:** Cortisol concentrations in tears, saliva, and blood [least square means (LSM) ± standard error (SE); ng/ml] in 48 sheep on days 1 to 3 (before the treatment phase); comparisons among sampling times (day 1, 2, 3) within matrices (Tukey test).

**Cortisol concentrations (LSM ±SE) in ng/ml**	**Sampling times (day)**		
	**1**	**2**	**3**
Tears	2.2 ± 0.2	1.9 ± 0.2	2.0 ± 0.2
Saliva	2.8 ± 0.2	2.7 ± 0.2	2.8 ± 0.2
Blood	44.7^a^ ± 1.7	34.8^b^ ± 1.7	36.6^b^ ± 1.7

LSM with different superscripts indicate significant differences between the corresponding mean values (*p* ≤ 0.01).

### 3.2 Dorsal recumbency vs. standing

On the morning of day 4, before the start of the treatment phase, the COT and COB were higher in sheep restrained in dorsal recumbency compared with measurements made 4 h later (Min 0) in standing sheep (*p* < 0.0001; Table 2).

**Table 2:** Cortisol concentrations in tears and blood [least square means (LSM) ± standard error (SE); ng/ml] in 48 sheep in dorsal recumbency in the morning of day 4, and in standing sheep (day 4, Min 0) at the start of the treatment phase; comparison between sampling times (day 4 in the morning and day 4 Min 0 of treatment phase) within matrices (Tukey test).

**Cortisol concentrations (LSM ±SE) in ng/ml**	**Position during sampling**	
	**Dorsal recumbency**	**Standing**
Tears	2.2^a^ ± 0.2	0.9^b^ ± 0.1
Blood	34.8^a^ ± 1.7	17.9^b^ ± 1.7

LSM with different superscripts indicate significant differences between the corresponding mean values (*p* ≤ 0.0001).

### 3.3 Cortisol concentration during the treatment phase

**Figure 3:**
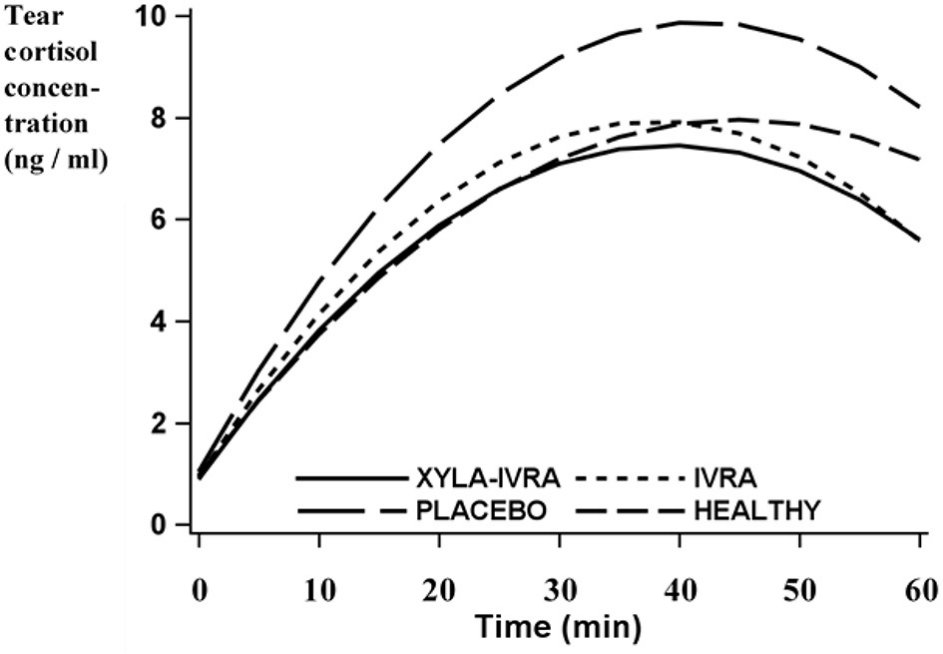
Tear cortisol concentrations (least square means; ng/ml) estimated with polynomials of second degree in 12 HEALTHY sheep and in 36 sheep with *dermatitis interdigitalis contagiosa* undergoing different pain management protocols (XYLA-IVRA = sedation and local anesthesia, *n* = 12; IVRA = local anesthesia, *n* = 12; PLACEBO, *n* = 12) during the treatment phase in a tilt squeeze chute: Min 0: sheep standing in a tilt squeeze chute and receiving drugs according to group allocation; Min 10: placement and restraint of the sheep in dorsal recumbency; Min 20: application of IVRA or placebo according to group allocation; Min 30: actual or sham claw treatment; Min 40: resumption of standing position; Min 60: sampling in group pen; tears were sampled after each procedure.

During the treatment phase, the COT (Figure 3) and COB [Figure 3 in Rachidi et al. (15)] had an analogous pattern across all treatment groups. The lowest COT was observed at the beginning of the treatment phase (min 0) and increased in all groups up to the time of claw treatment (min 30; Supplementary Table 2). The maximum COT (YMAX) in the PLACEBO group (9.9 ± 0.8 ng/ml [least square means ± standard error]) tended to be higher than the maximum COT in the XYLA-IVRA group (7.3 ± 0.8 ng/ml [least square means ± standard error]; *p* = 0.07; Table 3). At all time points during the treatment phase, the COT in the XYLA-IVRA group was numerically lower than in the PLACEBO group. After claw treatment, the COT began to decline (Table 3), but was still higher at the end of the treatment phase (min 60) than at the beginning (min 0; *p* < 0.0001; Supplementary Table 2). The area under the curve of COT and the TMAX did not differ among the groups (Table 3). With the exception of the IVRA group, TMAX of COT occurred after TMAX of COB [Table 4 in Rachidi et al. (15)]. The median difference between the TMAX of COT and COB was 6.1 min (range: 0.1–9.2 min).

**Table 3:** Area under the curve [least square means ± standard error (SE); ng/ml] calculated using quadratic regression, maximum cortisol concentration (least square means ± SE; ng/ml), and time point of the maximum concentration (± SE; Min) in tears in 12 HEALTHY sheep and in 36 sheep with dermatitis interdigitalis contagiosa undergoing different pain management protocols (XYLA-IVRA = sedation and local anesthesia, *n* = 12; IVRA = local anesthesia, *n* = 12; PLACEBO, *n* = 12) during the treatment phase in a tilt squeeze chute, and comparison between the groups (Tukey test).

**Tear cortisol concentrations**	**Area under the curve (ng/ml × Min ±SE)**	**Maximum cortisol concentration (ng/ml ±SE)**	**Time point of maximum concentration (Min ±SE)**
XYLA-IVRA	5.5 ± 0.6	7.26 ± 0.8	41.0 ± 3.6
IVRA	6.2 ± 0.6	8.52 ± 0.8	37.2 ± 3.7
PLACEBO	7.2 ± 0.6	9.95 ± 0.8	44.8 ± 3.7
HEALTHY	6.3 ± 0.5	8.26 ± 0.7	46.9 ± 3.4
*p*_XYLA − IVRA vs. IVRA_	0.78	0.65	0.88
*p*_XYLA − IVRA vs. PLACEBO_	0.13	**0.07**	0.87
*p*_XYLA − IVRA vs. HEALTHY_	0.69	0.78	0.63
*p*_IVRA vs. PLACEBO_	0.61	0.59	0.49
*p*_IVRA vs. HEALTHY_	0.99	0.99	0.23
*p*_PLACEBO vs. HEALTHY_	0.67	0.42	0.97

*P*-values that show a trend toward a significant difference (*p* ≤ 0.10) are in bold.

**Table 4:** Cortisol concentrations in tears, saliva, and blood [least square means (LSM) ± standard error (SE); ng/ml] in 37 sheep on days 4 (before the treatment phase) and days 5 and 6 (both after treatment), and within-group comparisons (Tukey test).

**Cortisol concentration in ng/ml (LSM ±SE)**	**Sampling times (days)**			**Comparison of time points within groups**		
	**4**	**5**	**6**	*p*_4vs.5_	*p*_4vs.6_	*p*_5vs.6_
Tears	2.5 ± 0.1	3.3 ± 0.3	2.6 ± 0.2	**0.02**	0.85	**0.10**
Saliva	2.7 ± 0.2	3.3 ± 0.2	2.6 ± 0.2	**0.08**	0.95	**0.05**
Blood	34.2 ± 1.8	39.6 ± 1.8	35.8 ± 1.8	**0.01**	0.74	**0.09**

*P*-values that are significant or show a trend toward significance (*p* ≤ 0.10) are in bold.

### 3.4 Cortisol concentration after the treatment phase

Tear cortisol concentrations were significantly elevated on day 5 compared with day 4 (before the treatment phase) and showed a tendency to decline on day 6 (*p* = 0.1). Similarly, there was a trend toward a higher COS on day 5 compared with the previous day and a significant decrease on day 6 (*p* = 0.05). The COB curve had a similar pattern, with significantly higher concentrations on day 5 and a downward trend on day 6, similar to the changes observed in the COT and COS (Table 5).

**Table 5:** Phenotypic correlations [r_p_ ± standard error (SE)], estimated with three-trait models, between the cortisol concentrations of blood, saliva and tears in the study sheep on days 1 to 6.

**Phenotypic correlations [rp ±standard error (SE)] between cortisol concentrations of**	**Sampling times (days)**					
	**1**	**2**	**3**	**4**	**5**	**6**
Blood:tears	0.46 ± 0.1	0.61 ± 0.1	0.69 ± 0.1	0.44 ± 0.1	0.60 ± 0.1	0.50 ± 0.2
Blood:saliva	0.51 ± 0.1	0.71 ± 0.1	0.58 ± 0.1	0.38 ± 0.2	0.61 ± 0.1	0.45 ± 0.2
Tears:saliva	0.87 ± 0.04	0.86 ± 0.05	0.67 ± 0.1	0.57 ± 0.1	0.88 ± 0.05	0.67 ± 0.1

The samples on day 4 were collected before the treatment phase and the samples on days 5 and 6 after the treatment phase. Days 1 to 4, *n* = 48; days 5 and 6, *n* = 37. All correlations are different from 0 with *p* ≤ 0.01.

### 3.5 Correlation of cortisol concentration in tears, saliva and blood

The COT, COS, and COB were positively and significantly correlated during the adaptation phase and on the days following. The correlation between the COT and COS was particularly close (Table 5). Positive correlations occurred between the COB and COT, the AUC of COB and AUC of COT, the maximum COB and maximum COT, and the time points of the maximum cortisol concentration during the treatment phase (Tables 6, 7).

**Table 6:** Phenotypic correlations ± standard error, estimated with two-trait models per minute, between the cortisol concentrations in tears and blood in sheep during the treatment phase in a tilt squeeze chute (day 4, min 0 to 60).

**Correlation ±standard error**	**Sampling times (min)**					
	**0**	**10**	**20**	**30**	**40**	**60**
Blood—tears	0.63 ± 0.1	0.59 ± 0.1	0.58 ± 0.1	0.67 ± 0.1	0.71 ± 0.09	0.77 ± 0.08

All correlations are different from 0 with *p* ≤ 0.01.

**Table 7:** Phenotypic correlations ± standard error, estimated with two-trait models, between the cortisol concentrations in blood and tears between the area under the curve (AUC), maximum cortisol concentration (YMAX), and time point of maximum concentration (TMAX) in sheep during the treatment phase in a tilt squeeze chute (day 4, min 0 to 60).

**Correlation ±standard error**	**AUC**	**YMAX**	**TMAX**
Blood—tears	0.65 ± 0.1	0.72 ± 0.09	0.71 ± 0.09

All correlations are different from 0 with *p* ≤ 0.01.

## 4 Discussion

### 4.1 Detection of cortisol in tears and saliva

The COS, COT, and COB did not differ between healthy sheep and those chronically affected with DINCO during the 4-day adaptation phase. DINCO is a common claw disease and the most frequent cause of lameness in sheep worldwide. It has a major impact on animal health, production, and welfare (44, 45), but its effects on the COT and COS have not been investigated (PubMed search on December 18, 2025, keywords: cortisol, sheep, lameness, claw disease). In agreement with our findings in the adaptation phase, other studies showed that the COB did not differ between healthy and diseased sheep, and the COS did not differ between healthy and lame cattle (46, 47). To date, data on COT have only been reported for healthy sheep (20), and studies in other animal species, including cattle, horses, dogs, seals, and humans, have not compared COT in healthy and lame individuals (32, 35, 48, 49). Our findings support the use of saliva and tears as alternative matrices for assessing acute stress responses in sheep, regardless of whether they have DINCO.

In our study, the COT and COS followed a pattern similar to the COB, albeit at markedly lower levels, reaching only about 5%−10% of the COB. In sheep, the proportion of free to total cortisol in blood ranges from about 10% at baseline to 25% at peak concentrations after various stress models and ACTH stimulation (39, 42, 50). Other studies in sheep, cattle and horses have also shown that saliva and tears predominantly contain free, biologically active cortisol (21, 34), which explains the lower COT and COS relative to COB observed in our study.

Correlations between cortisol concentrations in our study were positively and significantly across all three matrices, particularly between the COT and COS. Comparable or even higher correlations between the COB and COS have previously been reported in sheep (26, 50, 51). A study comparing cortisol concentrations in blood with those in tears, saliva, milk, and feces in cattle found that correlations were consistently stronger between the COT and COS than between the COB and COT or COB and COS (21). In the present study, a key strength compared with previous studies (26, 34, 36, 48, 52, 53) is that the reported correlations were derived from a study design including a comparatively large number of animals (*N* = 48) and repeated measurements (*N* = 554) at short intervals over several consecutive days. This approach enabled the assessment of both intra- and inter-individual variability in cortisol concentrations (54). The applied statistical analysis was based on a multi-trait model that incorporated the same linear mixed models used for the estimation of least-squares means. Phenotypic correlations were estimated from the residual effects of the multi-trait model, thereby accounting for and removing the influence of treatment and study group effects. This methodological framework strengthens the validity of the observed associations and supports the conclusion that both the COS and COT are suitable alternatives to COB to detect an acute stress response in sheep.

### 4.2 Detection of acute stress response in tears

#### 4.2.1 Daily restraint

Similar to the COB, the COT of sheep in a standing position (day 4, treatment phase, min 0) was significantly lower compared with earlier sampling of sheep in dorsal recumbency (day 4, morning). Restraint in dorsal recumbency represents a substantial stressor in sheep (14). Our findings illustrate that daily restraint and sample collection elicit an acute stress response, which can be reliably confirmed by measuring the COT.

#### 4.2.2 Comparison of cortisol concentration in tears of different pain control groups during the treatment phase

During the treatment phase, sheep in all pain control groups had COT dynamics that mirrored those of COB, with a trend to significant differences between the XYLA-IVRA and PLACEBO groups. To date, the COT has only been investigated in healthy sheep and cattle, in which similar patterns were reported (20, 21, 52). To the authors' knowledge, the COT has not been used to compare different stress and pain management protocols (PubMed search on December 18, 2025; keywords: cortisol, tear, stress management). Our findings suggest that the COT reflects an acute stress response as well as its magnitude.

However, although the patterns of COT and COB were similar, the COT followed COB with an average time delay of 6.1 min. A similar delay was observed in healthy sheep and cattle, where the COT increased 6–11 min after an increase in the COB (20, 21, 52), and similar time spans were described for the COS relative to COB (26, 50). The time delay results from the diffusion of free cortisol from blood into tears (32) and must be carefully considered when designing studies and interpreting results.

The differences between the XYLA-IVRA and PLACEBO groups were less pronounced for the COT than for the COB. Although a time delay has previously been discussed as a possible explanation for discrepancies in the COS (50), our design with frequent and closely spaced sampling intervals eliminated this factor. Instead, the effect of xylazine, which stimulates α2-adrenoceptors and therefore reduces tear secretion (55), should be considered. Free cortisol may have been concentrated in tears, artificially elevating the COT in the XYLA-IVRA group. Based on the present results, it cannot be conclusively determined whether xylazine attenuates the stress response itself or, due to its diverse systemic effects, alters the cortisol concentration in blood and tears. However, a stress-reducing effect of xylazine across several species has been demonstrated during various procedures [claw trimming and treatment (14, 15, 43, 56), surgical procedures (57–61), transport (13)] that evaluated not only cortisol but also behavioral and physiological parameters. Although the potential concentrating effect of xylazine on COT or COS has not been addressed in the literature (PubMed search on December 18, 2025; keywords: cortisol, tears, saliva, xylazine), it warrants careful evaluation in future studies, as xylazine may confound tear cortisol measurements.

#### 4.2.3 Adaptation phase and memory of stress

Unlike the COB, the COT and COS did not decline during the 4 days preceding the treatment phase to the levels measured in standing animals on day 4 at min 0. This shows that, as in blood, no adaptation to daily handling procedures was detectable in these two matrices, and adaptation would probably have taken a longer period of time (20). On day 5, 1 day after the treatment phase, the sheep had increased cortisol concentrations across all matrices in response to stress exposure on day 4, indicating a stress memory effect (15). Likewise, the COB was significantly higher on day 1 than on the following days. This pattern was only evident numerically in the COT, but not in the COS. Even on the day after the stress model, the increase in cortisol concentration was significant in tears, similar to blood, and only slight differences occurred in saliva. Therefore, the COT may be a sensitive marker, capable of reflecting small increases in cortisol concentration not expressed by the COS.

In relative terms, the COT and COS represented a smaller proportion of COB on day 1 than on the following days, suggesting that the proportion of free cortisol in blood was higher than in tears and saliva on that day (34, 42). This also may have been due to catheter manipulation, triggered by the memory of catheter placement the previous day (15, 62, 63), leading to a transient increase in free cortisol in the blood. In contrast, saliva and tear samples were collected before catheter handling on day 1, when cortisol levels had not yet increased to the same extent, and concentrations were comparable to those on subsequent days. Catheter handling is not inherently painful (51), and thus, the sheep responded comparably across all matrices to the applied stressors^1^ on the following days. These findings suggest that the COB is affected not only by catheter placement but also by stress memory on the following day. The concentration dynamics observed for the COT and COS in the present study warrant further investigation.

### 4.3 Critical appraisal of the study protocol

#### 4.3.1 Assessment of stress and pain

In the present study, both healthy and chronically lame sheep with painful DINCO lesions (10, 15, 44, 47), responded to non-nociceptive stress and pain with an increase in COB, COT and COS, with the magnitude of stress response depending on the treatment. Pain represents a stressor that elevate the cortisol concentration in sheep and cattle (59, 64–66). Therefore, determination of the cortisol concentration is an indirect but established method of assessing pain-induced stress (59, 67–69). However, due to the complex interaction between stress and pain (67, 70), cortisol concentration alone do not allow discrimination between non-nociceptive stress and pain. In the present study, such differentiation was of limited relevance, as both non-nociceptive stressors, e.g. due to unfamiliar environment or isolation, and pain, e.g. due to the claw treatment or defensive movements during restraint, compromise animal welfare (23, 71). Future studies should include additional physiological and behavioral traits if a distinction between the different stressors is required (59, 67, 68).

#### 4.3.2 Avoiding invasive blood collection

Sufficient amounts of saliva and tears were obtained for analysis at all time points, and sampling was feasible without adverse health effects, such as gingivitis or conjunctivitis, even though sampling was done repeatedly over several days and multiple times per day. In contrast, blood collection requires venipuncture (53) or the use of a catheter, such as one placed in the jugular vein (13), which demands experience and sterile conditions, and may affect cortisol concentration due to procedure-associated pain and stress (51, 62, 63). Catheterization of the jugular vein also increases the risk of thrombophlebitis (15, 19), which is why we also had to exclude measurements from 11 sheep starting on day 5 in our study. Thrombophlebitis is further associated with pain, stress, and potentially serious complications such as endocarditis (20, 72–74). Additionally, the inflammatory changes associated with thrombophlebitis can lead to alterations in the blood profile (75), which may impair the availability and analysis of free cortisol (76, 77). Since an influence on the secondary matrices could not be ruled out (76, 78), COT and COS from sheep with thrombophlebitis were also excluded from the analysis. While repeated blood sampling can cause adverse effects (21), saliva and tears were collected over long periods without health complications. Moreover, the use of saliva and tears does not pose the risk of catheter-associated injuries caused by herdmates (20), and caretakers familiar with the animals can collect samples, thereby reducing stress (26). The ease of use makes the collection of tears and saliva particularly suitable for field studies or large-scale experiments where catheterization is impractical (79). It is also animal welfare-friendly and supports efficient experimental design. However, to avoid bias in cortisol measurements, animals should be free of ocular or oral inflammation and injuries that could lead to blood contamination, and saliva samples should not contain feed residue or regurgitated cud.

#### 4.3.3 Measuring biologically active cortisol

The same immunoassay kit was used for measuring free cortisol, the biologically active fraction (42), in saliva and tears, and the total cortisol concentration in blood. This method is widely used and particularly suitable for determining acute stress responses through the measurement of free cortisol (21, 51). Direct comparability between matrices could be further enhanced by determining both free and total cortisol in blood (42). While immunoassays are generally recommended to be validated for both species and matrix (39), the assay we used has been successfully applied to saliva and tears in sheep and cattle (20, 21). Thus, the methodological approach of our study reflected an acute stress response by determining biologically active cortisol. Future studies may complement this design by matrix-specific validation and the determination of free and total cortisol, further strengthening direct comparability between matrices.

#### 4.3.4 Field study design and restraint

Our study combined the advantages of a field setting, including optimal housing conditions, familiar caretakers, and a practice-relevant stress model, with the rigor of a randomized, controlled, prospective, and blinded design. The latter included experienced and specially trained veterinarians, standardized procedures, and statistical adjustments, ensuring that potential environmental influences known to significantly affect stress responses (4, 80, 81) were minimized. Practical stress models reflect realistic management situations and incorporate cognitive evaluation of stimuli by animals, which ultimately shapes the cortisol response (82). As a result, our study provides robust and practice-oriented insights into stress responses during sample collection, restraint, and claw treatment in a tilt squeeze chute.

In the present study, sheep of different ages were included. Within each study group, the age of the four sheep was matched as closely as possible in order to minimize within-group age-related variability. Across study groups, however, age differences were present, reflecting the availability of animals at the time of enrollment of sheep into the respective study group. Previous studies in younger sheep, goats, cattle, and sows have reported higher (83, 84), lower (85, 86) or comparable (87, 88) cortisol concentrations than in older individuals. The individual stress response vary considerably between animals and is influenced, among other factors, by prior experience as well as individual learning capacity and adaptability to the stressor (89, 90). As detailed information on the complete experiential history of each animal is typically unavailable, it is not possible to reliably predict individual stress appraisal or to fully disentangle animal-related factors from environmental influences. To consider the effects of both individual animal-related factors as well as environmental effects on the cortisol concentration, the study group was included as fixed effects in all statistical models. This approach accounts for potential influences at the group level and reduces potential confounding (91–93), likewise applied in previous stress-related studies (94–99). We therefore recommend this approach for future studies with comparable experimental designs.

Saliva samples were collected using an established, well-tolerated method that uses the natural chewing reflex of small ruminants (100). Our method of collecting tear samples was shown to be successful in cattle (21). Nevertheless, both tear and saliva collection required slight head restraint. Because restraint is a stressor in calves and sheep (14, 101), sampling in this study was performed when sheep were already restrained in dorsal recumbency. To minimize additional head restraint during the treatment phase, sampling was limited to blood and tears. This represents a limitation of the present study, as COS could not be assessed during the treatment phase. Future studies may adapt the protocol to include simultaneous blood, tear and saliva sampling to enable a more comprehensive comparison of COT, COS and COB. Our results indicate that sheep tolerate slight head restraint without a stress response, whereas dorsal recumbency constitutes a substantial stressor, as confirmed by higher cortisol responses during dorsal recumbency compared with standing restraint. Under these circumstances, head restraint did not represent an additional significant stressor. Therefore, in situations where restraint is unavoidable for the applied stress model, the collection of saliva and tear samples causes only negligible stress and can be considered non-invasive (102). However, for future studies without mandatory restraint, contactless monitoring methods may be preferable. Approaches such as infrared thermography (101) or new biosensor technologies, including contact lens-based cortisol sensors (25), show promising ways to integrate biomarker analysis into intelligent, non-invasive systems for monitoring wellbeing.

#### 4.3.5 Sample contamination

Daily physical examination throughout the study ensured that none of the sheep had injuries or inflammatory changes, such as gingivitis or conjunctivitis, which may have altered the composition of saliva or tears or caused blood contamination of these matrices. Blood contamination distorts cortisol concentrations in saliva and tears (39, 49), while ocular inflammation, such as conjunctivitis, is painful and alters tear composition (103, 104). Gentle handling reduces the risk of defensive movements and sampling injuries (105). Animals with ocular or oral lesions should be excluded because pain elevates cortisol levels (67).

To minimize feed contamination of saliva, animals were briefly housed without access to feed before sample collection. Feed residues compromise assay accuracy and must be avoided (39). Short-term feed withdrawal does not cause stress provided that housing is adequate (106). In contrast, prolonged feed deprivation induces hunger, which itself is a stressor (3, 107). Additionally, in sheep, regurgitated cud may contaminate saliva during extended sampling protocols (20). Therefore, exclusive use of tears may be preferable during intensive short-term sampling periods.

Finally, immediate freezing of samples ensured stability and prevented bacterial growth. Freezing halts bacterial metabolism and disrupts mucins in saliva that otherwise complicate processing (39). Saliva hosts abundant bacterial communities with limited antimicrobial proteins (37), whereas tears contain antimicrobial components (108). Studies in humans demonstrated that the COS declines when samples are stored at room temperature (109). Thus, immediate freezing is essential for both matrices, though tears may be inherently less susceptible to microbial degradation.

## 5 Conclusion

Saliva and tears are reliable, minimally invasive collected alternatives to blood for assessing acute stress in healthy and chronically lame sheep. Cortisol in both matrices reflected an acute stress response. The time delay observed in the COT compared with the COB was consistent with previously reported values for saliva, allowing for a consistent sampling schedule in future studies. Tear and saliva collection proved to be minimally invasive, repeatable, and practical, consistently providing sufficient material for analysis without impacting health or stimulating stress. However, head restraint was required, making tear and saliva collection best suited for studies where head restraint has no impact or is part of a procedure. Our findings also support further development of contactless sampling techniques. Determining the COT may be slightly more practical because tears pose a lower risk of contamination and harbor fewer bacteria than saliva. Moreover, the suitability of frequent saliva sampling needs to be determined, particularly concerning the effects of rumination. Our protocols and results contribute to the refinement of stress detection methods, support animal welfare, and offer alternative options for future research. They provide practical applications in animal husbandry and monitoring strategies for individuals unable to express stress verbally.

## Data Availability

The original contributions presented in the study are included in the article/Supplementary material, further inquiries can be directed to the corresponding author.

## Footnotes

1 The following stressors were applied: Min. 0: restraint in chute, intramuscular administration of drugs according to group allocation. Min. 10: dorsal recumbency. Min. 20: RIVA or placebo. Min. 30: actual or sham claw treatment. Min. 40: resumption of standing position. Min. 60: manual restraint and sampling in the group pen.
