# Supplementary material for: Tears and saliva as alternative matrices for minimally invasive assessment of acute stress and pain in sheep
Source: Front Vet Sci. 2026 Jan 28;13:1719442. doi: 10.3389/fvets.2026.1719442 (PMC12890685; doi:10.3389/fvets.2026.1719442)
Supplement: Supplementary file 1 [file Data_Sheet_1.pdf]

frontiers

Supplementary Material

1 Supplementary Tables

Supplementary Table 1: Cortisol concentrations in tears, saliva and blood (least square means (LSM) ± standard error (SE); ng/ml) in healthy (N = 12) and diseased (N = 36) sheep on days 1 to 3 (before the treatment phase). Healthy and diseased sheep are compared within time points, and time points are compared within groups (Tukey test)

**Cortisol concentrations in ng/ml (LSM ± SE)**		**Sampling times (day)**			**Comparison of time points within groups**		
		**1**	**2**	**3**	**$p_{1\ vs.\ 2}$**	**$p_{1\ vs.\ 3}$**	**$p_{2\ vs.\ 3}$**
**Tears**	Healthy	1.9 ± 0.3	2.0 ± 0.4	2.3 ± 0.4	0.99	0.92	0.98
	Diseased	2.3 ± 0.2	1.8 ± 0.2	2.0 ± 0.2	0.2	0.74	0.95
	P-value	0.31	0.52	0.46			
**Saliva**	Healthy	2.5 ± 0.3	2.6 ± 0.4	2.9 ± 0.4	0.99	0.89	0.87
	Diseased	2.9 ± 0.2	2.7 ± 0.2	2.8 ± 0.2	0.76	0.89	0.99
	P-value	0.34	0.72	0.82			
**Blood**	Healthy	43.1[a] ± 3.3	37.3[b] ± 3.3	38.2[b] ± 3.3	0.44	0.76	0.99
	Diseased	45.3[a] ± 2.1	34.1[b] ± 2.1	36.1[b] ± 2.1	**<0.0001**	**0.002**	0.85
	P-value	0.62	0.39	0.56			

P - values that are significant or show a trend toward significance ($p \leq 0.10$) are in bold.

Supplementary Table 2: Tear cortisol concentrations (least square means (LSM) ± standard error (SE); ng/ml) in 12 HEALTHY sheep and in 36 sheep with *dermatitis interdigitalis contagiosa* undergoing different pain management protocols (XYLA-IVRA = sedation and local anesthesia, n = 12; IVRA = local anesthesia, n = 12; PLACEBO, n = 12) during the treatment phase. Sampling was done after each procedure. The concentrations were compared within groups at each time point, and within groups between Min. 0 and Min.60, and between Min. 20 and Min. 40 (Tukey test)

**Tear cortisol concentrations in ng / ml (LSM ± SE)**	**Sampling times (min)**						**P - value**	
	**0**	**10**	**20**	**30**	**40**	**60**	$p_{0\ vs.\ 60}$	$p_{20\ vs.\ 40}$
**XYLA-IVRA**	0.89 ± 0.15	3.81 ± 0.33	5.88 ± 0.56	7.09 ± 0.69	7.45 ± 0.75	5.60 ± 0.94	**<0.01**	**<0.01**
**IVRA**	0.93 ± 0.17	4.13 ± 0.34	6.36 ± 0.56	7.62 ± 0.69	7.91 ± 0.75	5.57 ± 0.94	**<0.01**	**<0.01**
**PLACEBO**	1.04 ± 0.16	4.76 ± 0.33	7.47 ± 0.56	9.17 ± 0.69	9.86 ± 0.75	8.21 ± 0.94	**<0.01**	**<0.01**
**HEALTHY**	0.97 ± 0.15	3.73 ± 0.33	5.80 ± 0.56	7.18 ± 0.69	7.87 ± 0.75	7.17 ± 0.94	**<0.01**	**<0.01**
$p_{XYLA\text{-}IVRA\ vs.\ IVRA}$	0.99	0.90	0.93	0.95	0.97	1.00		
$p_{XYLA\text{-}IVRA\ vs.\ PLACEBO}$	0.89	0.20	0.20	0.16	0.12	0.22		
$p_{XYLA\text{-}IVRA\ vs.\ HEALTHY}$	0.98	0.99	0.99	0.99	0.98	0.64		
$p_{IVRA\ vs.\ PLACEBO}$	0.97	0.55	0.50	0.40	0.27	0.22		
$p_{IVRA\ vs.\ HEALTHY}$	0.99	0.83	0.89	0.97	1.00	0.63		
$p_{PLACEBO\ vs.\ HEALTHY}$	0.99	0.15	0.17	0.20	0.26	0.86		

The following stressors were applied in the treatment phase: Min. 0: restraint in chute, intramuscular administration of drugs according to group allocation. Min. 10: dorsal recumbency. Min. 20: IVRA or placebo. Min. 30: actual or sham claw treatment. Min. 40: resumption of standing position. Min. 60: manual restraint and sampling in the group pen
